# Contrast-enhanced mammography-guided biopsy in a prone position in the diagnosis of breast cancer: technical parameters and clinical outcomes

**DOI:** 10.1186/s13244-025-02163-7

**Published:** 2025-12-17

**Authors:** Giulia Morano, Federica Cicciarelli, Giuliana Moffa, Giacomo Bonito, Veronica Rizzo, Alessandro Calabrese, Francesca Galati, Federica Pediconi

**Affiliations:** Department of Radiological, Oncological and Pathological Sciences, Sapienza, University of Rome, Policlinico “Umberto I”, Rome, Italy

**Keywords:** Contrast-enhanced mammography, CEM-guided biopsy, Prone table, MRI-guided biopsy, Breast cancer

## Abstract

**Objectives:**

To evaluate the technical parameters and clinical outcomes of contrast-enhanced mammography (CEM)-guided biopsy for diagnosing breast cancer in patients with suspicious lesions showing post-contrast enhancement on CEM but undetectable on standard digital mammography (DM) or ultrasound (US).

**Materials and methods:**

A prospective study was conducted on 36 patients referred for CEM-guided biopsy based on suspicious (BI-RADS 4-5) enhancing-only lesions detected during previous CEM examinations and/or contrast-enhanced breast MRI (CE-MRI). Procedures were performed on a dedicated prone table using a vacuum-assisted biopsy device. Effectiveness parameters included success rate (lesion enhancement, diagnostic material collection, and correct clip positioning), procedure time, average glandular dose (AGD), compression force, and complication rate.

**Results:**

From January to November 2024, 36 patients underwent CEM-guided biopsy, with a success rate of 97.2% (35/36). The median procedure time was 29 min. The AGD was 0.88 mGy (range 0.5–1.4 mGy, SD ± 0.22). The average compression force was 4.94 kg (range 2–7 kg, SD ± 1.1). Of the 35 lesions biopsied, 20 (57.1%) were masses and 15 (42.9%) non-mass enhancements, with a mean lesion size of 13.2 mm. Breast lesions were classified as BIRADS 4a (10/35), BIRADS 4b (5/35), BIRADS 4c (8/35), and BIRADS 5 (12/35). Histopathological findings showed 57.1% (20/35) of lesions were malignant. Lesion classification included 5.7% (B1), 34.3% (B2), 2.9% (B3), 31.4% (B5a), and 25.7% (B5b).

**Conclusion:**

CEM-guided biopsy is an effective and accessible technique for targeting enhancing-only breast lesions, offering advantages over MRI-guided biopsy in terms of time, cost, and patient comfort.

**Critical relevance statement:**

Contrast-enhanced mammography-guided biopsy is an effective and accessible technique for targeting enhancing-only breast lesions, offering advantages over MRI-guided biopsy in terms of time, cost, and patient comfort.

**Key Points:**

- Contrast-enhanced mammography (CEM)-guided biopsy has recently gained traction as a reliable alternative to MRI for targeting enhancing-only lesions.
- This study explores the clinical implementation of CEM-guided biopsy in a prone position in our university hospital, assessing its efficacy, safety, and diagnostic accuracy.
- CEM-guided biopsy is a promising technique for the precise targeting of breast lesions, with advantages over MRI-guided biopsy.

**Graphical Abstract:**

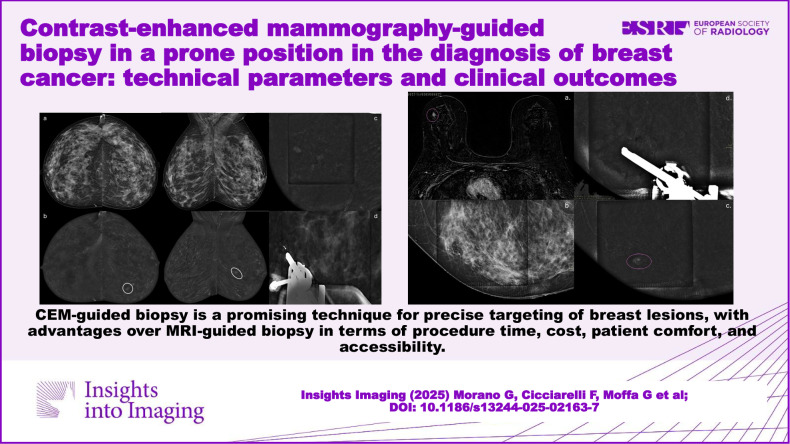

## Introduction

Contrast-enhanced mammography (CEM) represents a newcome, promising technique in breast imaging and CEM-guided biopsy—particularly when performed in a prone position—is emerging as a pivotal innovation in this field. Currently, ultrasound (US)-guided biopsy is the preferred method in clinical practice due to its good efficiency and accessibility and low overall costs. However, it has notable limitations, particularly in detecting architectural distortions or microcalcifications, for which stereotactic biopsy is generally preferred [1–4].

Certain breast lesions, however, need the injection of intravenous contrast to be made visible, as occurs during magnetic resonance imaging (MRI) or CEM examinations. While targeted US can localize 46–71% of such lesions [5], the subset of “enhancing-only” lesions has historically required MRI guidance for percutaneous biopsy [5–7]. Despite its diagnostic utility, the use of MRI-guided biopsy is limited by patchy availability, high costs, and contraindications like claustrophobia or metallic implants [8, 9].

CEM-guided biopsy has recently gained traction as a reliable alternative to MRI for targeting enhancing-only lesions. This technique employs vacuum-assisted biopsies (VAB) facilitated by a stereotactic CEM add-on unit integrated with the mammography system [4, 10–12]. Studies have shown that CEM-guided biopsy offers a diagnostic performance comparable to that of MRI-guided procedures [8], while generating significant advantages such as shorter procedure times, reduced costs, broader availability, and fewer contraindications [13, 14]. Moreover, a higher overall preference of patients toward CEM vs. breast MRI was previously demonstrated, mainly due to exam duration, greater comfort, lower noise, and lower rates of anxiety linked to the procedure [15].

As a hybrid imaging modality, CEM combines the morphological details of standard mammography with functional information derived from the use of intravenous iodinated contrast agents. This dual capability enables a precise evaluation of both lesion morphology and tumor angiogenesis, outperforming traditional techniques like digital mammography (DM) and US, as well as digital breast tomosynthesis (DBT) [16–20]. With sensitivity akin to MRI [15] and strong histopathological correlation [21, 22], CEM is increasingly used for problem-solving, pre-operative staging, neoadjuvant therapy monitoring, and post-treatment evaluation, among others [17, 23–25].

In the last 5 years, CEM has been introduced and employed increasingly widely in our university hospital, mainly as a problem solver or as an additional diagnostic tool in women at higher-than-average risk. This study explores our experience with the clinical implementation of CEM-guided biopsy, assessing its efficacy, safety, and diagnostic accuracy. By analyzing procedural metrics, histological outcomes, costs, and patient factors such as complication rates, we aim to delineate the role of CEM in modern breast cancer diagnosis. Through this comprehensive evaluation, our research seeks to establish the practical advantages of CEM-guided biopsy and how it can integrate into routine clinical practice.

## Materials and methods

### Study design and population

This prospective study adhered to Good Clinical Practice guidelines and received the approval of our Institutional Review Board. Each patient signed a written informed consent to be included in the study. The study included 50 patients referred for CEM-guided biopsy based on suspicious enhancing lesions (BI-RADS categories 4 and 5) detected during previous CEM examinations and/or breast contrast-enhanced MRI, which were not reliably identified at conventional breast imaging techniques (digital mammography or ultrasound). After a targeted second-look ultrasound confirmed the absence of a detectable correlate, these patients were considered eligible for CEM-guided biopsy according to the current standard of care between January 2024 and November 2024. In this study, a previous CEM was performed based on several clinical indications, including better evaluation of dense breast tissue or parenchymal distortion. It was also selected for patients with contraindications to MRI or those for whom MRI was less suitable (ex., claustrophobia). Additionally, some high-risk patients preferred CEM for follow-up due to its shorter examination time and greater comfort compared to MRI.

In case of MRI-detected lesions, patients proceeded directly to CEM-guided biopsy. Exclusion criteria were: suspected or confirmed pregnancy; ongoing lactation; history of allergic reactions to iodinated contrast agents or to local anesthetic; abnormal coagulation parameters (PT, PTT, and INR); impaired renal function according to the guidelines of the European Society of Urogenital Radiology (ESUR) for radiologic contrast agent [26]; presence of breast implants, to avoid the known artifact caused by implants in recombined images; core needle biopsy (CNB) or VAB performed in the 14 days before the CEM procedure to prevent potential biases related to complications of these procedures (such as hematomas); incomplete medical records.

### CEM-guided biopsy procedure

The study was approved by the local Ethics Committee with the reference number RM124190DFS08850, and before starting the procedure, patients were thoroughly informed about the examination, including the possible adverse reactions to the contrast agent administered. Written informed consent was obtained from all patients, and a comprehensive medical history was taken.

VAB was carried out using an 11-gauge semi-automatic needle (Mammotome; Ethicon Endo-surgery) under mammography guidance. All examinations were performed on a low-dose digital mammography unit capable of performing CEM (Giotto Class with CESM; IMS Giotto).

The procedure followed the principles of stereotactic guidance, with the additional step of contrast agent injection to enhance lesion visibility.

The biopsy procedure began by selecting the optimal approach, based on the location of the lesion on previous CEM images and the patient’s body type. The choice of biopsy approach—medial or lateral—was based on the breast’s compressed thickness: a vertical approach was used for breasts thicker than 3 cm, while a horizontal approach was preferred for thinner breasts. The shortest distance from the skin to the lesion was determined to guide the needle placement. The biopsy table is equipped with a dedicated opening for the breast containing the target lesion. This design ensures that only the affected breast lies below the table, properly aligned with the mammography unit and the biopsy equipment.

Before starting the procedure, a non-ionic iodinated contrast agent (Table 1) was power-injected intravenously at a dose of 1–1.5 mL/kg body weight. Contrast agent administration was followed by a 20-mL saline flush at the same rate. Approximately 2 min after contrast agent administration, the breast was compressed using a biopsy window, while the patient was already positioned prone on the biopsy table, and a mammogram was acquired to confirm the accurate centering of the area of interest within the biopsy window, according to the distances measured during the pre-procedural setup.

**Table 1:** Technical characteristics of the CEM procedure

	Mean	Minimum	Maximum	Standard deviation
Lesion size (mm)	13.2	4	60	± 15.3
AGD (mGy)	0.88	0.5	1.4	± 0.22
Compression force (kg)	4.94	2	7	± 1.1
Injection flow rate (mL/s)	2.76	2.5	3.0	± 0.25
Volume injected (mL)	103.1	90	120	± 6.8

Contrast medium	Frequency (%)
Omnipaque 350 mg/mL	3 (8.3%)
Iomeron 400 mg/mL	12 (33.3%)
Iopamiro 370 mg/mL	8 (22.2%)
Ultravist 370 mgI/mL	5 (13.9%)
Optiray 350 mg/mL	8 (22.2%)

Breast density	Frequency (%)
A	6 (16.7%)
B	15 (41.7%)
C	9 (25%)
D	6 (16.7%)

BPE	Frequency (%)
Minimal	11 (30.6%)
Mild	16 (44.4%)
Moderate	6 (16.7%)
Marked	3 (8.3%)

*AGD* average glandular dose, *BPE* background parenchymal enhancement

Once the lesion was localized and compressed, dual-energy imaging at 0°, +15°, and −15° was performed. A low-energy exposure (22–35 kVp) and a high-energy exposure (40–49 kVp) were acquired serially. The recombined images showing enhanced areas were automatically reconstructed by a post-processing dedicated software.

**Fig. 1:**
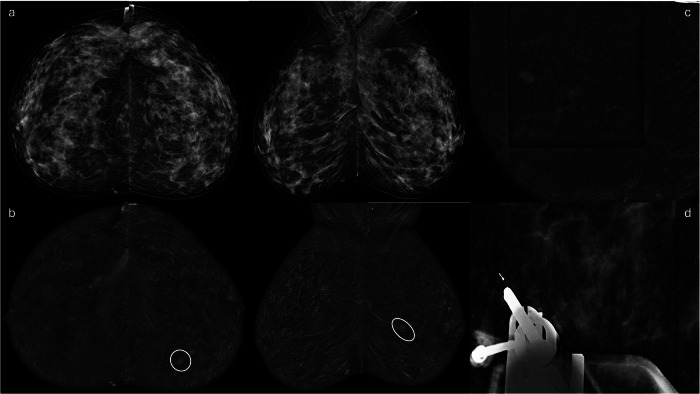
**a** During the screening mammography of an asymptomatic patient, some microcalcifications are found in the left upper outer quadrant. **b** The subsequent CEM highlighted a mass enhancement of 7 mm between the left inner quadrants; no enhancement was detected in the site of the calcifications. **c** A left CEM-guided biopsy was performed, with horizontal needle approach, followed by (**d**) post-biopsy marker placement. On histological examination, the lesion was NST-IDC (G1)

**Fig. 2:**
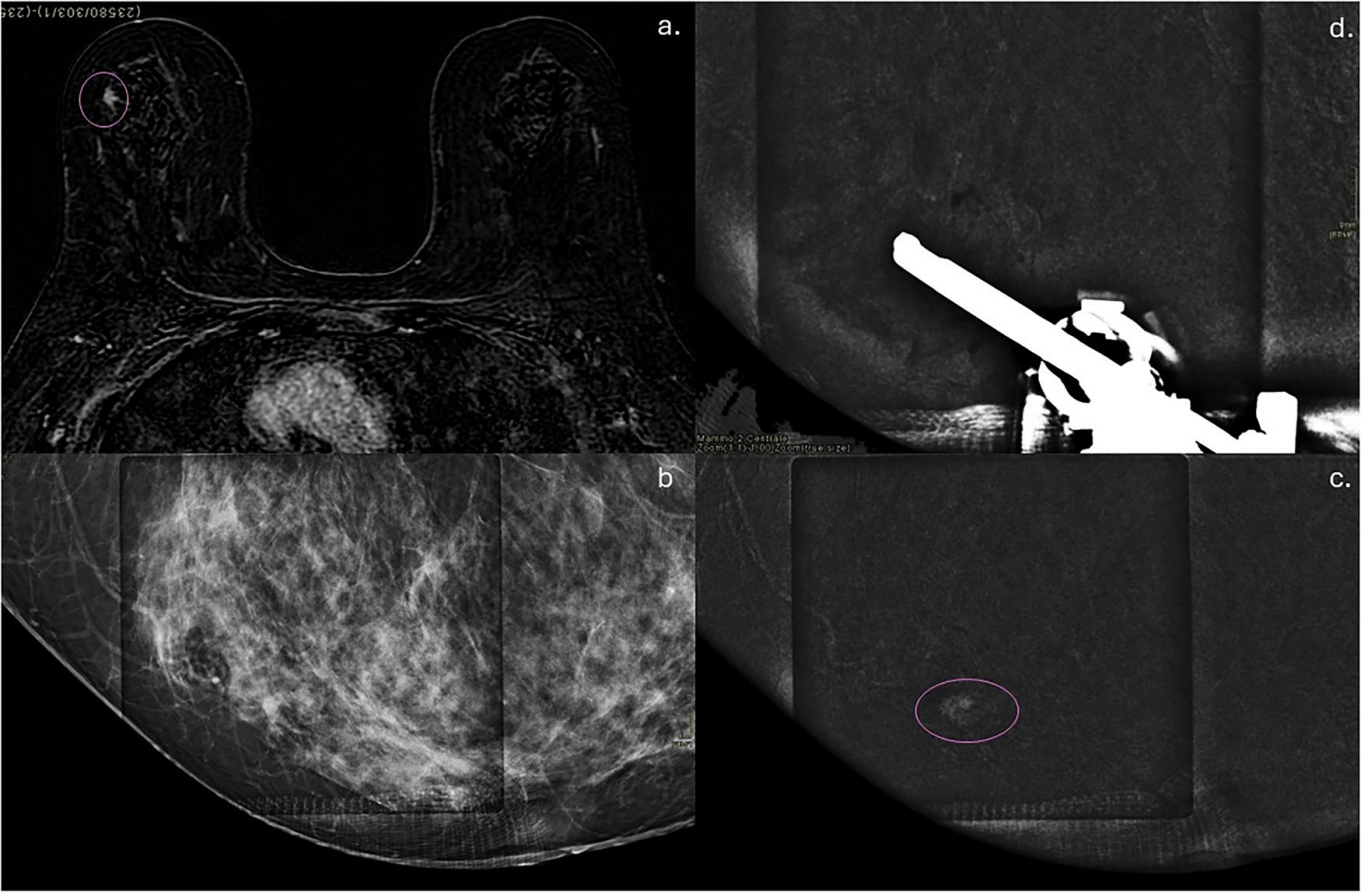
**a** MRI examination of a high-risk patient: a 6 mm mass enhancement highlighted in the upper outer quadrant of the right breast; **b**–**d** CEM-guided biopsy was performed with a horizontal approach, and a corresponding mass enhancement was confirmed. On histological examination, the lesion was NST-IDC

Using a computerized coordinate system, the machine directed the biopsy needle toward the target. After antisepsis and local anesthesia, the needle holder was pushed through the breast tissues until it reached the target. A final imaging check confirmed that the lesion remained correctly positioned in the biopsy window. Once the target was verified, the needle was fired, and samples were collected with multidirectional biopsies in a complete clockwise rotation. At least 12 samples were collected for each patient (Figs. 1, 2).

Finally, a stereotactic marker was placed to facilitate future localization of the biopsy site, and an additional mammographic acquisition was performed to confirm the correct marker placement.

### Data collection and statistical analysis

Clinical data of the included patients, such as age, breast density, and BPE, as well as information about the suspicious lesions, including enhancement features and size, were acquired from medical records and recorded.

Breast density was assessed using a dedicated software (Tiziano Software, IMS Giotto) on low-energy images and classified according to the American College of Radiology (ACR) BI-RADS® lexicon for CEM [27] in four categories: ACR BI-RADS A (almost entirely fatty breasts), ACR BI-RADS B (scattered areas of fibro-glandular density), ACR BI-RADS C (heterogeneously dense), and ACR BI-RADS D (extremely dense).

BPE was assessed visually on the diagnostic recombined image and graded into the following four categories: minimal, mild, moderate, and marked, according to the ACR BI-RADS® lexicon [27].

Enhancing lesions were classified as mass or non-mass enhancement (NME), according to the ACR BI-RADS lexicon for CEM [27–29]. Masses were characterized in terms of shape (round, oval, or irregular), margins (circumscribed or non-circumscribed, irregular or spiculated), internal enhancement pattern (homogeneous, heterogeneous, or rim-enhanced), and lesion conspicuity (low, moderate, or high). Distribution, internal enhancement pattern, and lesion conspicuity further characterized NME.

A BIRADS score was assigned for each lesion and collected.

The BPE and BI-RADS scoring were evaluated independently by two radiologists, each with 20 and 10 years of experience in breast imaging, respectively. To ensure accuracy, a subset of cases was independently reviewed by a second radiologist, and any discrepancies were resolved by consensus.

Procedural characteristics, including duration of the procedure, average glandular dose (AGD), and average compression force, were collected for each examination.

Moreover, we collected the histopathological results from VAB or surgical specimens (for patients who underwent surgery).

All data were acquired anonymously.

Patient age and duration of the procedure were presented as median and range. Other continuous variables were presented as mean, range, and standard deviation. Categorical variables were reported using counts and percentages.

## Results

**Fig. 3:**
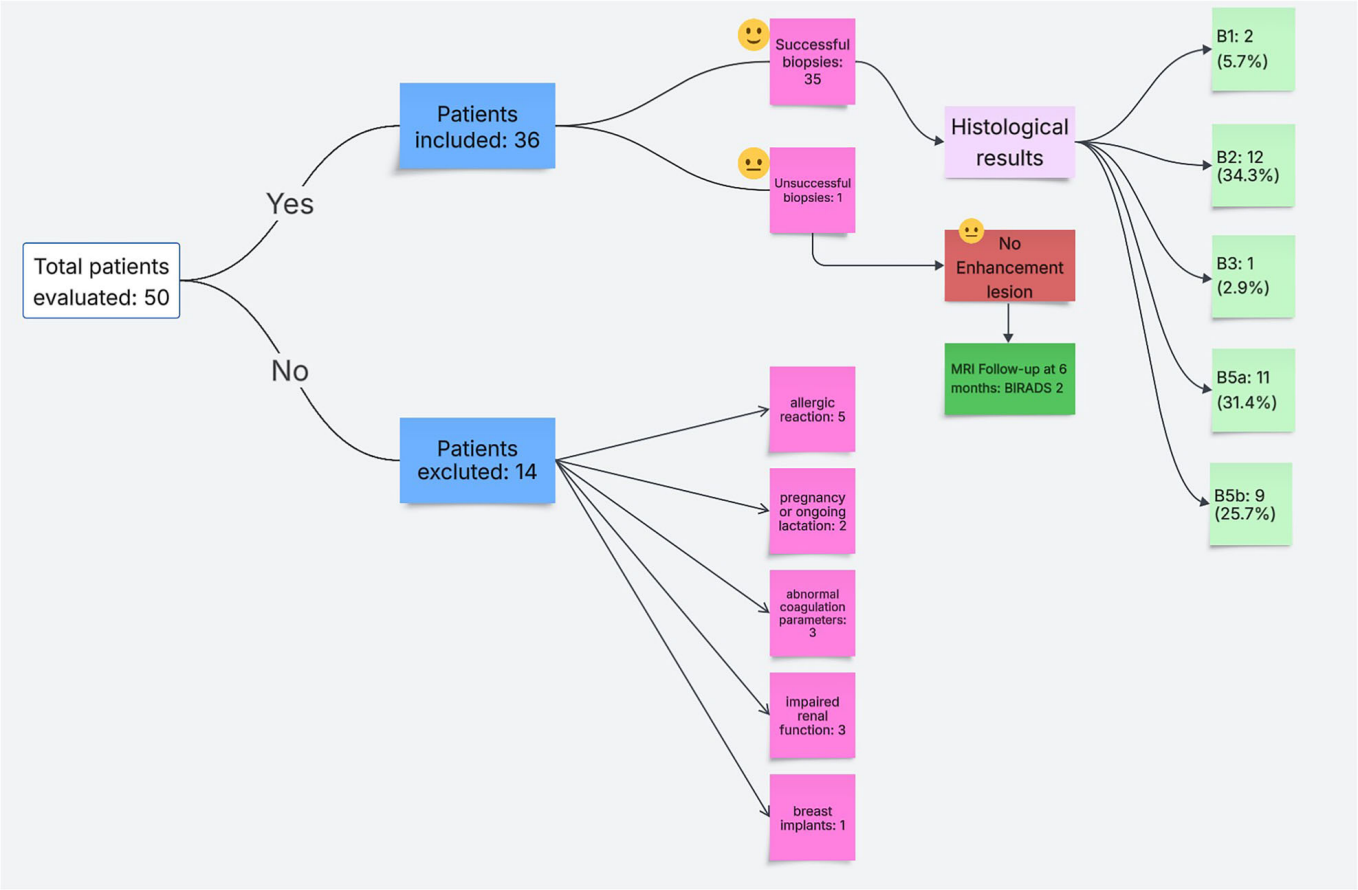
Flowchart of the patient selection process

From January 2024 to November 2024, 36 out of 50 patients were included in the evaluation and referred for CEM-guided biopsy, while 14 patients were excluded. The procedure was completed in 35 out of 36 patients, yielding a success rate of 97.2% (full lesion targeting). In one case, no enhancement was observed during the CEM-guided biopsy procedure, prompting a referral for close MRI follow-up (see Fig. 3 for details).

Among the 36 patients included in the study, 27 (75%) were referred for CEM-guided biopsy based on suspicious findings identified on CEM, while 9 patients (25%) had suspicious findings initially detected on breast MRI, which had been performed as part of high-risk screening or follow-up protocols (e.g., in patients with a family history of breast cancer or other risk factors). In one of these cases, the procedure was not successful, resulting in 35 completed biopsies.

The median age of the patient group was 54.5 years (range 40–80 years). The median duration of the entire procedure (from positioning to dressing) was 29 min (range 19–52 min).

The mean AGD for a single acquisition during CEM-guided procedures was 0.88 mGy (range 0.5–1.4 mGy, SD ± 0.22). The mean compression force was 4.94 kg (range 2–7 kg, SD ± 1.1). Additional procedure characteristics are summarized in Table 1.

Breast density was classified as ACR A in 6/36 (16.7%) patients, ACR B in 15/36 (41.7%), ACR C in 9/36 (25%), and ACR D in 6/36 (16.7%) patients. The BPE after contrast injection was minimal in 11/36 (30.6%), mild in 16/36 (44.4%), moderate in 6/36 (16.7%), and marked in 3/36 (8.3%). Enhancement was observed in 35/36 patients. Among the 35 suspicious lesions, 20 were masses (57.1%) and 15 were NME (42.9%). The mean size of the suspicious lesion was 13.17 mm (range 4–60 mm, SD ± 15.3). Breast lesions were classified as BIRADS 4a (10/35), BIRADS 4b (5/35), BIRADS 4c (8/35), and BIRADS 5 (12/35).

Of the 35 lesions successfully biopsied, 20 (57.1%) were found to be invasive or in situ breast cancer. Specifically, 2/35 (5.7%) lesions were B1, 12/35 (34.3%) were B2, 1/35 (2.9%) was B3, 11/35 (31.4%) were B5a, and 9/35 (25.7%) were B5b. Details about the biopsied lesions are summarized in Table 2.

**Table 2:** Characteristics of the breast lesions

	Value
Success rate	35/36 (97.2%)
Lesions with contrast enhancement	35/36 (97.2%)
Enhancement	*N* = 35
Mass enhancement	20 (57.1%)
Non-mass enhancement	15 (42.9%)
BIRADS classification	*N* = 35
B1	2 (5.7%)
B2	12 (34.3%)
B3	1 (2.9%)
B4	0 (0.0%)
B5a	11 (31.4%)
B5b	9 (25.7%)
Histology result	*N* = 35
Normal tissue	2 (5.7%)
Adenosis/sclerosis	9 (25.7%)
Hyaline fibrosis	1 (2.9%)
Fibrocystic mastopathy	1 (2.9%)
Fibroadenoma	1 (2.9%)
Radial scar	1 (2.9%)
DCIS	11 (31.4%)
IDC	7 (20.0%)
ILC	2 (5.7%)

*DCIS* ductal carcinoma in situ, *IDC* invasive ductal carcinoma, *ILC* invasive lobular carcinoma

At the end of the procedure, the correct placement of clip is confirmed, and no cases of clip migration were observed.

After the procedure, 3/35 (8.6%) patients experienced complications (a small hematoma for each patient). No adverse reactions to the contrast agent were recorded.

## Discussion

This study investigated the use and reliability of CEM-guided biopsy in a prone position for the evaluation and precise targeting of enhancing-only breast lesions, demonstrating its high success rate (97.2%). This excellent result is comparable to that of MRI-guided biopsy (87–98%) [30] and underscores the feasibility of CEM-guided biopsy as a concrete alternative to MRI-guided procedures, offering comparable diagnostic performance with several advantages, including a shorter procedure time, potentially lower costs, and greater accessibility.

Although we have not performed a formal cost-effectiveness analysis, our experience suggests a potential economic advantage for CEM over breast MRI. In our institution, operating under the Italian National Health System (Sistema Sanitario Nazionale), breast MRI reimbursement is approximately 230 euros, compared to 100 euros for CEM. Future comprehensive cost-effectiveness studies should include equipment, personnel, and operational costs as well as long-term healthcare outcomes, in order to establish the true economic impact across different healthcare settings.

Additionally, the difference in examination time should be considered, as it may contribute to lower overall costs. The median procedure time was 29 min, which is notably shorter than MRI-guided biopsy, which typically takes 60–70 min [31, 32]. This reduction in procedure time likely contributes to increased patient comfort and satisfaction. Other authors have observed a general preference for CEM over MRI examination, due to its shorter duration, lower noise levels, and less discomfort [19, 31, 33]. The same is probably true for biopsy procedures as well. Additionally, the prone position significantly contributed to procedure success by minimizing biopsy-related stress. Since patients cannot see the operative field or biopsy needle, anxiety and distress are considerably reduced. This approach also prevented procedural interruptions, contributing to the high success rate observed.

While the seated approach has been widely adopted and well-documented, prone positioning remains largely underexplored, so far. To our knowledge, the only comprehensive evaluation of this technique was conducted by Nori et al [34] based on 37 CEM-guided biopsies using a prone table. According to the results of this study, as well as our preliminary experience, the prone position seems to provide significant advantages over the traditional sitting position, including a considerable reduction in vasovagal reactions and consequent fainting, a well-known complication in the sitting position [31, 34], and improved overall patient comfort. The main limitations of the prone approach are the necessity for specialized equipment (a prone table system) and limited experience.

The sitting position remains the most commonly used and well-documented approach, with the significant advantage of easy patient positioning, but preliminary results suggest that prone positioning offers superior patient tolerance and procedural feasibility.

Comparing success rates between positioning approaches, Nori et al [34] achieved a 97.3% conclusive histological diagnosis rate in prone positioning, compared to the 95.4% reported for seated CEM-guided biopsies [4]. These results are consistent with ours and suggest that prone positioning may offer a comparable or slightly superior success rate, probably due to reduced patient movement, which may enhance targeting accuracy.

Among the 35 successfully biopsied lesions studied, 57% were found to be invasive or in situ breast cancer, which is consistent with previously reported cancer detection rates of MRI-guided biopsies (18–61%) [30, 35, 36]. In particular, 31.4% were DCIS and 25.7% invasive carcinoma (NST-IDC or ILC). These findings support the good diagnostic accuracy of CEM-guided biopsy in terms of breast cancer detection.

An important advantage of CEM-guided biopsy is its ability to assess lesion morphology on low-energy images, which resemble diagnostic mammograms and facilitate effective lesion localization, using anatomical landmarks from mammography images. This approach enabled accurate targeting even in challenging cases, such as those involving NME (42.9%) or small masses (28.6%). The use of non-contrast low-energy images for lesion identification during CE studies is particularly valuable if compared to breast MRI, where pre-contrast sequences are often insufficient for cancer identification, particularly in detecting microcalcifications and architectural distortions that are crucial for targeting [30, 35].

Typically, the target site for MRI- or CEM-guided biopsy is only the enhancing lesion. Using CEM-guided biopsy, it is possible to visualize associated imaging features, such as microcalcifications or architectural distortions, which can serve as anatomical landmarks to aid localization [30, 35, 36].

Our success rate was significantly facilitated by the morphologic features of suspicious lesions at pre-contrast CEM acquisitions, which enabled partial reconstruction by comparing recombined and low-energy diagnostic CEM images with DBT images. This approach allowed the identification of landmarks for biopsy guidance and facilitated the comparison of suspicious findings observed in the absence of contrast administration. This strategy has also been previously reported by Alcantara et al [4] and Kornecki et al [37].

Despite these undeniable advantages, CEM-guided biopsy procedures seem to present some limitations, primarily related to BPE. In our study, the success rate of CEM-guided biopsy was not affected by BPE. Possible explanations include the low breast density of the majority of patients included in the study and the relatively small sample size. According to a recently published research by Moffa et al [20], the BPE observed on CEM examination is strongly associated with breast density at DM, being higher in women with dense breasts. On the contrary, it is currently believed that BPE levels on breast MRI are not clearly influenced by breast composition. Nonetheless, in a study comparing BPE on CEM and breast MRI, Sogani et al [38] have demonstrated that breast density assessed at low-energy CEM images and breast MRI images were comparable and that both were predictors of BPE levels. This discrepancy may be attributed to the lack of systematic methods for the quantification of breast density and BPE levels [38].

BPE can compromise the diagnostic quality of CEM-guided biopsy as well, particularly in patients with extremely dense breasts (ACR D). In our study, breast density was classified as ACR D only in a minority of patients (16.7% of the total population), and only 8.3% of cases showed marked BPE. Even if the size of this subsample was small, the success of CEM-guided biopsy was not affected by the presence of BPE, suggesting that CEM guidance remains a strong tool even in the case of high BPE levels. Similar conclusions can be deduced from the results of a pioneering study about CEM-guided biopsy by Alcantara et al [4].

Regarding safety, complications linked to the procedure were minimal; only 8.6% of patients experienced early hematomas, all of which resolved without severe sequelae. This outcome is not surprising, since small post-procedure hematomas are common but not usually worrisome [35, 39]. No allergic reactions were recorded among our study population, further supporting the nowadays well-established safety of CEM. Moreover, various iodinated contrast agents were employed during our research, with no significant differences in diagnostic outcomes, indicating that different iodinated contrast agents can be used interchangeably in CEM-guided biopsy, offering flexibility in clinical practice.

As is known, one of the main disadvantages of CEM is the radiation dose. However, the AGD values measured during the CEM-guided procedures included in the study were within safe limits and lower than those reported in the literature [4, 40]. A recent study comparing CEM guidance to stereotactic and DBT-guided biopsy [41] has demonstrated that CEM-guided and stereotactic biopsy show similar AGD values per acquisition and per procedure, suggesting that CEM-guided biopsy does not pose a significant increase in radiation exposure compared to traditional imaging techniques. The dosimetric findings reported in the study (mean AGD for a single CEM-biopsy acquisition = 1.48) are comparable to those observed in our investigation. The same research has shown that DBT guidance was more dose-efficient per procedure, probably due to the lower number of acquisitions required, but it lacks information about lesion enhancements. The main limitations of our study include the small sample size and the single-center nature, which could both affect the generalizability of the results. Larger, multicenter research is necessary to validate our findings and further refine the procedural outcomes of CEM-guided biopsy.

## Conclusion

In conclusion, CEM-guided biopsy is a promising technique for the precise targeting of breast lesions, offering several advantages over MRI-guided biopsy in terms of procedure time, cost, patient comfort, and accessibility. Notably, when performed using a prone table and stereotactic approach, it improves patient tolerance by enhancing comfort, minimizing the stress linked to the biopsy itself and reducing the risk of fainting—a possible complication in seated positions—without increasing radiation dose. This study adds to the growing body of evidence suggesting that CEM-guided biopsy is not only a valid alternative to MRI-guided biopsy but could potentially replace it in selected clinical scenarios, pending further confirmatory studies.

## Data Availability

The datasets analyzed during the current study are available from the corresponding author upon reasonable request. Access to the data may be subject to restrictions due to privacy considerations. Specific details regarding the nature of the datasets and how they can be accessed will be provided upon request to ensure compliance with relevant guidelines and policies.

## Abbreviations

ACR: American College of Radiology
AGD: Average glandular dose
BPE: Background parenchymal enhancement
CEM: Contrast enhancement mammography
DBT: Digital breast tomosynthesis
DCIS: Ductal carcinoma in situ
DM: Mammography
ILC: Invasive lobular carcinoma
MRI: Magnetic resonance imaging
NME: Non-mass enhancements
NST-IDC: Invasive carcinoma of no special type-invasive ductal carcinoma
US: Ultrasound
VAB: Vacuum-assisted biopsy
